# A cluster of inhibitory residues in the regulatory domain prevents activation of the cystic fibrosis transmembrane conductance regulator

**DOI:** 10.1016/j.jbc.2025.108460

**Published:** 2025-03-26

**Authors:** Min Wu, Yawei Xiong, Mengyuan Cao, Yunqi Zhi, Yan Jin, Yizhen Huang, Jeng-Haur Chen

**Affiliations:** 1 College of Life Sciences, Zhejiang Normal University, Jinhua, Zhejiang Province, China; 2 Department of Applied Science, College of William & Mary, Williamsburg, Virginia, USA; 3 Department of Gynecology and Obstetrics, Jinhua People's Hospital, Jinhua, Zhejiang Province, China; 4 School of Information Engineering, Jinhua Polytechnic University, Jinhua, Zhejiang, China; 5 Departments of Internal Medicine, Roy J. and Lucille A. Carver College of Medicine, University of Iowa, Iowa City, Iowa, USA; 6 Howard Hughes Medical Institute, Roy J. and Lucille A. Carver College of Medicine, University of Iowa, Iowa City, Iowa, USA

**Keywords:** cystic fibrosis transmembrane conductance regulator, channel activation, PKA, protein phosphorylation and chloride channel

## Abstract

Activation of the cystic fibrosis transmembrane conductance regulator (CFTR) Cl^‒^ channel requires PKA phosphorylation at the regulatory (R) domain to relieve inhibition of ATP-dependent channel activity. This study aimed to identify the primary inhibitory site that prevents channel activation. CFTR mutants with deletion of residues 760 to 783 (ΔR_760–783_) elicited constitutive macroscopic and single-channel Cl^‒^ currents in the presence of ATP before PKA phosphorylation, suggesting that protein segment R_760–783_ in the R domain blocks CFTR activation. With the background of ΔR_760–835_, further deletion of R_708–759_ led to fully active channels in the presence of ATP, but the absence of PKA, suggesting that R_708–759_ prevents the activation of ΔR_760–835_-CFTR. R_760–783_ peptides were unstructured in buffered solutions in CD spectroscopy and the N771P mutation that interrupts the α-helix formation induced no apparent constitutive current before PKA phosphorylation. These data suggest that interpeptide interactions by α-helices likely contribute trivially to the blocking effect of R_760–783_. CFTR mutants with small deletions or alanine replacements containing any one of residues R^766^ and S^768^ in a PKA consensus sequence and M^773^ and T^774^ generated PKA-independent CFTR Cl^‒^ currents. Similarly, introducing the mutations Q767C or T774C into a control CFTR construct produced constitutive CFTR Cl^‒^ currents by positively charged 2-(trimethylammonium)ethylmethanethiosulfonate modification of target cysteines. Moreover, PKA-independent single-channel activity was evidently observed in R766K-, S768K-, and T774K-CFTR mutants. Therefore, the four residues, R^766^, S^768^, M^773^, and T^774^, may form an inhibitory module that precludes CFTR activation through side-chain interactions. This inhibitory mechanism might be emulated by other PKA-dependent proteins.

The cystic fibrosis transmembrane conductance regulator (CFTR) is an epithelial Cl^−^ channel, defective in the genetic disease cystic fibrosis (1). CFTR consists of two membrane-spanning domains (MSDs), two nucleotide-binding domains (NBDs), and a regulatory (R) domain (2). Cyclic AMP- or PKA-dependent phosphorylation of the R domain initiates activation of the CFTR Cl^−^ channel (3), after which cycles of ATP binding and hydrolysis at two ATP-binding sites located at the interface of the NBD1:NBD2 dimer control opening and closing (4) of a gate in MSDs (5) to regulate Cl^−^ flow through CFTR. When the R domain is dephosphorylated, CFTR activity ceases (3) even if the ATP concentration at the intracellular side of the channel is high (6). The cryo-EM structures of CFTR demonstrate that the unphosphorylated R domain may be present at the interface of two NBDs to prevent their dimerization (7), the essential conformation change that leads to channel opening (8). However, how the R domain blocks CFTR activity before PKA phosphorylation is still not well understood (9).

The boundary of the R domain from residues 634 to 836 (R_634–836_) has been defined by the deletion of a protein segment without apparently affecting ATP-dependent CFTR gating (10), whereas the ΔR_708–835_/S660A mutation that removes R_708–835_ and contains an additional S660A mutation largely eliminates PKA-dependent stimulation of channel activity (11, 12), creating a constitutively active channel in the presence of ATP alone (11) with low channel activity (12, 13). By various deletions among three short segments, R_708–759_, R_760–783,_ and R_784–835_, we recently found that R_815–835_ in R_784–835_ plays an essential role in phosphorylation-dependent stimulation of channel gating (13), which is autoinhibited by R_784–814_ (13). By contrast, previous work found that deletion of R_760–783_ alone generated PKA-independent constitutive CFTR Cl^−^ currents (14), suggesting that unphosphorylated R_760–783_ contains an inhibitory mechanism that prevents CFTR activation.

A further segment that may prevent CFTR activation is R_817–838_. Deletion of R_817–838_ generated a PKA-independent, constitutively active CFTR Cl^−^ channel (15). Conversely, the mutant that deletes R_784–835_ retained PKA-stimulated channel activity (14). Moreover, the significant activation of H950R/S768R-CFTR only requires ATP (16). These studies highlight the complexity of investigating CFTR activation as any modification of the R domain may not only remove inhibition of channel activation but also affect channel gating (12, 13, 15) and PKA-dependent regulation of channel activity (17).

Structurally, the R domain is mostly disordered (18, 19, 20). Phosphorylation decreases the α-helical propensity of R domain peptides (18, 19) and reduces interactions of peptide segments at several PKA phosphorylation sites, such as S^768^, with an isolated NBD1 peptide (19). A contrary finding indicates that phosphorylated R domain peptides show higher propensity of α-helix but less β-sheet and random coil than unphosphorylated peptides (20). Moreover, protein segments, such as R_825–843_ (7), R_763–777_ (19) and R_655–670_ (21) display prominent α-helical structure in the R domain. PKA phosphorylation is proposed to relocate the R domain from the interface of the NBD1:NBD2 dimer to the lasso motif, initiating channel gating by ATP (22).

Although large conformational changes of different domains accompany CFTR activation by PKA-dependent phosphorylation (22, 23), the major site that blocks CFTR activation in the R domain and the underlying mechanism remain unclear. The R domain may act like an inhibitory ball that occludes the channel pore (12), prevents NBD dimerization (7) or inhibits channel gating (7, 24).

It is of interest that S^768^ in the R_760–783_ segment is phosphorylated by PKA prior to other phosphorylation sites (17). In addition, the mutation S768A enhances cAMP-dependent activation of CFTR (25) and channel gating (17, 26). Moreover, interactions between S^768^ and intracellular loop 3 inhibit CFTR activity prior to PKA phosphorylation (16, 27). These data reveal an inhibitory role for S^768^ independent of R domain phosphorylation.

This study aimed to understand better how R_760–783_ and other segments block CFTR activation and the role of S^768^ in this inhibitory mechanism. We first investigated the PKA-independent channel activity of CFTR mutants with R_760–783_ deleted and then tested the effects of phosphorylation at S^768^ on the secondary structures of R_760–783_ peptides. Next, we explored the location of inhibitory residues and the role of their side-chain interactions in preventing channel activation. This study discovered a cluster of four residues that directly block CFTR activation by side-chain interactions.

## Results

### Protein segment R_760–783_ in the R domain prevents CFTR activation

**Figure 1:**
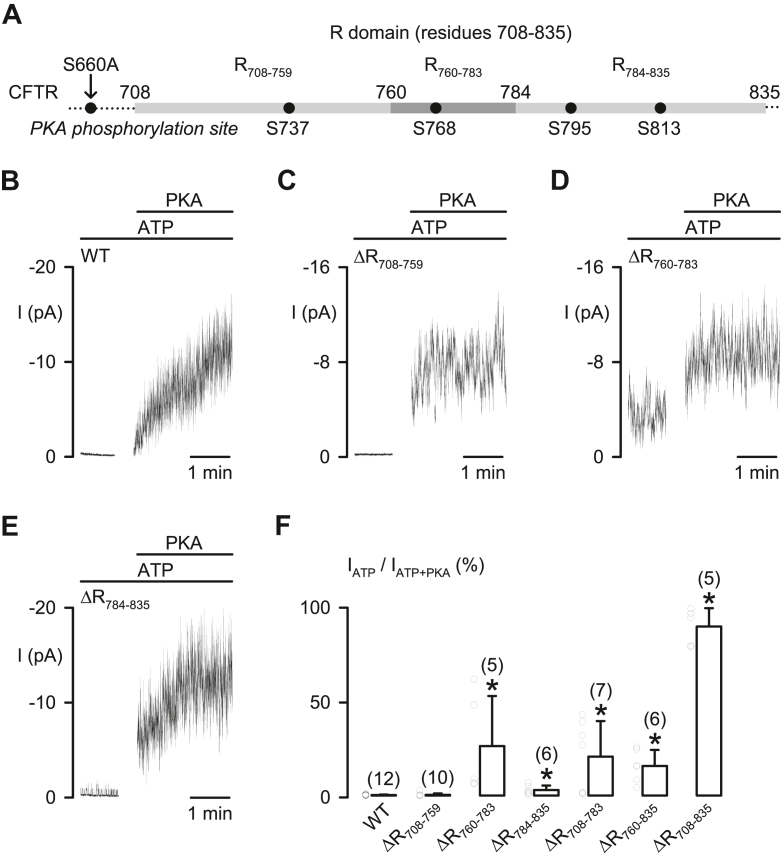
**R**_**760–783**_**isrequired to block CFTR activity**. *A*, the diagram shows the amino acid residues at the boundaries of the three designated R domain protein segments R_708–759_, R_760–783_, and R_784–835_. The positions of the four major PKA phosphorylation sites, S^737^, S^768^, S^795^, and S^813^, are indicated. All CFTR deletion mutants in this and other figures contain the S660A mutation. *B*–*E*, representative macroscopic CFTR Cl^−^ currents (I) of the R domain deletion mutants at 1 mM ATP before and after adding PKA (75 nM). *F*, fraction (%) of the CFTR Cl^−^ current in 1 mM ATP *versus* current in ATP + PKA (=100%) for all tested deletion mutants. Columns are means + SD with *circles* showing individual data points as independent biological replicates; numbers in *parentheses* indicate n; ∗*p* < 0.05 compared with WT CFTR as the control, one-way ANOVA with Dunn’s test. CFTR, cystic fibrosis transmembrane conductance regulator.

We began by investigating the role of three major protein segments, R_708–759_ (residues from position 708–759), R_760–783,_ and R_784–835_ (Fig. 1*A*), of the R domain in preventing CFTR activation, as described previously (13, 14). For comparison with previous work (13, 14), all R domain deletion mutations used in this study contain the mutation S660A (Fig. 1*A*).

In excised inside–out membrane patches at room temperature, the macroscopic Cl^−^ currents of wildtype CFTR (Fig. 1*B*) and the deletion mutant that removes R_708–759_ (ΔR_708–759_) (Fig. 1*C*) were trivial in the presence of ATP (1 mM) but were greatly increased after adding PKA (75 nM) into the bath solution (Fig. 1, *B* and *C*). By contrast, at 1 mM ATP, ΔR_784–835_-CFTR showed small currents (Fig. 1*E*), whereas all CFTR mutants with R_760–783_ deleted, including ΔR_760–783_-, ΔR_708–783_-, ΔR_760–835_-, and ΔR_708–835_-CFTR, exhibited constitutive CFTR Cl^−^ currents before PKA addition (Fig. 1, *D* and *F*). Our data are consistent with previous experiments performed at 37 °C (14). Thus, regardless of the recording temperature, the protein segment R_760–783_ markedly inhibits CFTR activation.

Among all mutants with R_760–783_ deleted, only ΔR_708–835_, not ΔR_760–783_, ΔR_708–783_, and ΔR_760–835_, lost most PKA-dependent stimulation of channel activity (Fig. 1*F*), suggesting that both R_708–759_ and R_784–835_ also inhibit CFTR activity. Therefore, the small constitutive current of ΔR_784–835_-CFTR (Fig. 1, *E* and *F*) may be caused by removing the inhibitory effects of R_784–835_ and/or by disturbing that of R_760–783_ (Fig. 1, *D* and *F*).

**Figure 2:**
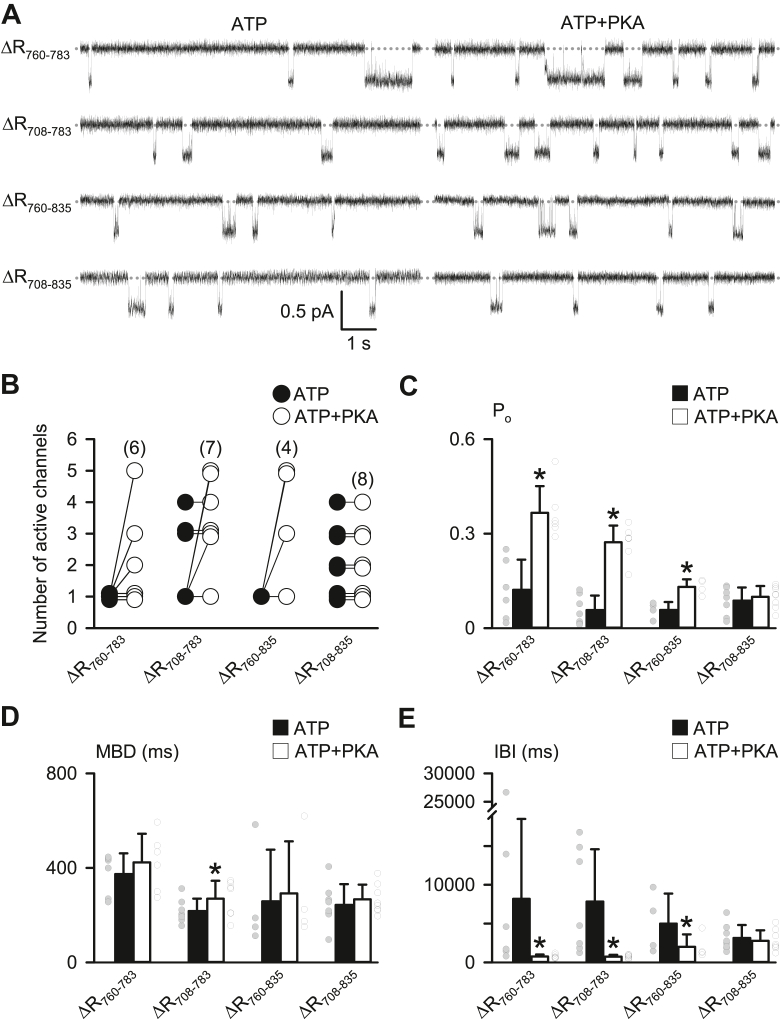
**Protein segments R**_**708–759**_**and R**_**760–783**_**inhibit CFTR activation but play a minor role in regulating channel gating before and after PKA phosphorylation**. *A*, representative recordings show the single-channel activity of ΔR_760–783_-, ΔR_708–783_-, ΔR_760–835_-, and ΔR_708–835_-CFTR in the presence of either ATP (1 mM) or ATP + PKA (75 nM). Each trace is 10 s long. The *dotted gray lines* indicate where channels are closed, and downward deflections represent channel openings. *B*, circles connected by *lines* show the number of active channels before (ATP) and after adding PKA (ATP+PKA) for each experiment of the indicated deletion mutants. For presentation purposes, some data points are positioned slightly higher or lower than the real integral number. *C*–*E*, effects of PKA phosphorylation on the open probability (P_o_), mean burst duration (MBD), and interburst interval (IBI) of the indicated deletion mutants. Columns are means + SD, and *circles* represent individual data points as independent biological replicates. Numbers in *parentheses* in *B* indicate n for *B*–*E*; ∗*p* < 0.05 compared with the data of the same deletion mutant at 1 mM ATP (*filled columns*), Student’s paired *t* test. CFTR, cystic fibrosis transmembrane conductance regulator.

Consistent with the macroscopic current data (Fig. 1*F*), all mutants with R_760–783_ deleted (Fig. 2*A*) exhibited single-channel activity at 1 mM ATP (*left tracings*, Fig. 2*A*), suggesting that R_760–783_ blocks CFTR activation by preventing the formation of active channels. The channel activity of ΔR_760–783_-, ΔR_708–783_-, and ΔR_760–835_-CFTR, but not ΔR_708–835_-CFTR, was further enhanced by PKA (*right tracings*, Fig. 2*A*). For different R domain deletion mutants, the frequency of observing two or more active channels before adding PKA was ΔR_760–783_-CFTR, 0/3 experiments; ΔR_708–783_-CFTR, 3/6 experiments; ΔR_760–835_-CFTR, 0/3 experiments; and ΔR_708–835_-CFTR, 5/5 experiments (Fig. 2*B*). These data suggest that R_708–759_ like R_760–783_ may interfere the formation of active channels.

To further examine and quantify this effect in the presence of multiple channels, we excluded the data from those membrane patches containing only one active channel (Fig. 2*B*) and then measured the ratio of the number of active channels before *versus* that after adding PKA. With an inhibitory segment R_784–814_ (13) present in both mutants, the ratio for ΔR_760–783_-CFTR was 0.34 ± 0.08 (N = 3), not statistically different from that of 0.62 ± 0.17 (N = 6) for ΔR_708–783_-CFTR (*p* > 0.05, one-way ANOVA with Dunn’s test). By contrast, the ratio for ΔR_760–835_-CFTR that contains R_708–759_ was 0.24 ± 0.04 (N = 3), smaller than that for ΔR_708–835_-CFTR 1.0 ± 0.0 (N = 5; *p* < 0.05, one-way ANOVA with Dunn’s test), supporting the notion that R_708–759_ prevents the formation of active channels of ΔR_760–835_-CFTR. However, the blocking effect of R_760–783_ is likely critical because no constitutive CFTR Cl^−^ current was induced by ΔR_708–759_ (Fig. 1*C*).

As R domain deletions may alter the PKA-dependent activation of CFTR, we further investigated the effects of PKA on the channel gating of each deletion mutant (Fig. 2, *C*–*F*). The open probability (P_o_) of all R domain deletion mutants in the presence of 1 mM ATP alone was small and similar to each other (*p* > 0.05, one-way ANOVA with Dunn’s test; *filled columns*, Fig. 2*C*), but, with the exception of ΔR_708–835_-CFTR, were markedly increased by PKA (Fig. 2*C*). Analyses of gating kinetics indicated that mean burst duration (MBD) values of the mutants were similar to each other before and after adding PKA (Fig. 2*D*), albeit that of ΔR_708–783_-CFTR was slightly increased by PKA (Fig. 2*D*). Consistent with the P_o_ changes of the R domain deletion mutants, values of interburst interval (IBI) were very long in the presence of ATP alone (*filled columns*, Fig. 2*E*) but markedly reduced by PKA, except for that of ΔR_708–835_-CFTR (Fig. 2*E*).

Thus, after R_760–783_ is deleted, the MBD of R domain deletion mutants is PKA independent, whereas the IBI is PKA dependent (Fig. 2, *D* and *E*). In contrast to the no change in the IBI of ΔR_708–835_-CFTR by PKA (Fig. 2*E*), adding PKA slightly decreased the IBI of ΔR_760–835_-CFTR (Fig. 2*E*), suggesting that the IBI shortening of ΔR_760–835_-CFTR is stimulated by PKA phosphorylation at R_708–759_.

Such a small IBI regulation by R_708–759_ was observed evidently only in the PKA-dependent activity of ΔR_760–835_-CFTR (Fig. 2*E*). The P_o_ and IBI values between ΔR_760–783_- and ΔR_708–783_-CFTR before or after adding PKA were not statistically different (*p* > 0.05, one-way ANOVA with Dunn’s test, Fig. 2, *C* and *E*), as low channel activity because of lacking R_815–835_ (13) and various deletion-caused structural constrains (10) may lead to large data variations in these two mutant CFTRs. It is of note that PKA-dependent increases in the number (Fig. 2*B*), P_o_ (Fig. 2*C*), and IBI (Fig. 2*E*) of ΔR_760–835_-CFTR may explain partial constitutive macroscopic currents of ΔR_760–835_-CFTR before PKA phosphorylation (Fig. 1*F*).

To better understand how R_760–783_ critically blocks CFTR activation, we explored whether the helical structure in this segment (19) may play an important role in blocking CFTR activation by generating α-helix-mediated interactions (15, 19). We further asked whether phosphorylation at the major PKA phosphorylation site S^768^ in this segment (Fig. 1*A*) may affect the formation of α-helix.

### Phosphorylation at S^768^ weakly reduces low-concentration trifluoroethanol-induced helix formation in R_760–783_ peptides

**Figure 3:**
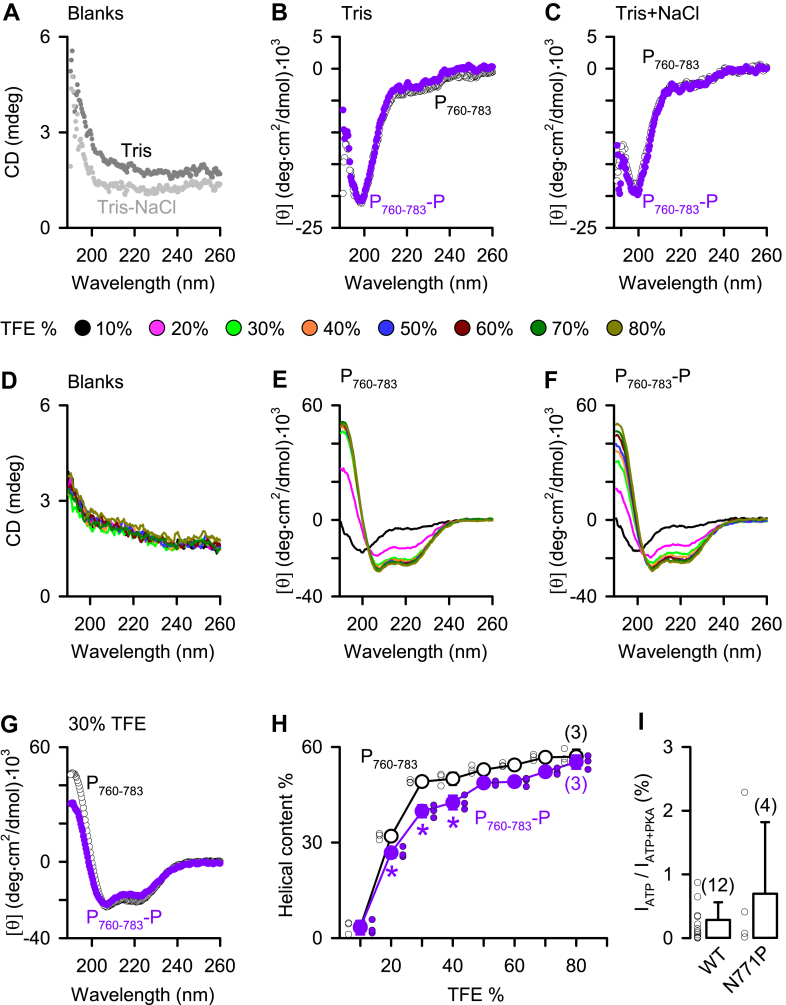
**Phosphorylation of S^768^ mildly reduces TFE-induced helix formation in R**_**760–783**_**peptides**. *A*–*C*, CD spectra of the buffer solutions containing only Tris or Tris + NaCl (*blanks*, *A*) and CD spectra of the Tris (*B*) or Tris + NaCl (*C*) buffered solution containing R_760–783_ peptides either unphosphorylated (R_760–783_, *open circles*) or phosphorylated at S^768^ (R_760–783_-P, *purple dots*). Similar results were obtained in three repeated experiments for each condition. In *B* and *C*, molar ellipticity ([θ]) is shown to correct CD spectra for the concentration of peptides. *D*–*F*, CD spectra of the Tris solutions with different concentrations of TFE only (*blanks*, *D*) or adding additional unphosphorylated (*E*) or phosphorylated (*F*) R_760–783_ peptides. *G*, CD spectra of the 30% TFE solutions containing unphosphorylated or phosphorylated R_760–783_ peptides. *H*, percentages of helical content for unphosphorylated and phosphorylated R_760–783_ peptides at different concentrations of TFE. Data are means ± SD (n = 3) with *small circles* indicating individual data points and some SD bars are smaller than symbol size; ∗*p* < 0.05 compared with the data of unphosphorylated R_760–783_ at the same percentage of TFE, one-way ANOVA with Holm–Sidak test. *I*, fraction (%) of the CFTR Cl^−^ current in 1 mM ATP *versus* current in ATP + PKA (=100%). Columns are means + SD with *circles* showing individual data points as technical replicates; numbers in *parentheses* indicate n. The data of WT CFTR were obtained from Figure 1*F*. CFTR, cystic fibrosis transmembrane conductance regulator; TFE, trifluoroethanol.

Under conditions of low background absorbance of the solvent Tris or Tris + NaCl buffer (Fig. 3*A*), CD spectrum experiments demonstrated that independent of phosphorylation status, R_760–783_ peptides (control, P_760–783_; phosphorylated at S^768^, P_760–783_-P) formed mostly random coil structures (Fig. 3, *B* and *C*). Using trifluoroethanol (TFE) to create a hydrophobic environment mimicking the condition of peptide–peptide interactions (28), both P_760–783_ and P_760–783_-P with similar low-background absorbance (Fig. 3*D*) showed CD spectrum characteristic of α-helix conformations at TFE concentrations ≥20% (Fig. 3, *E–H*), as seen in other helical peptides (29). Importantly, phosphorylation at S^768^ significantly decreased the α-helical propensity of P_760–783_ peptides at 20 to 40% TFE (Fig. 3, *G* and *H*). The data might suggest that R_760–783_ peptides are prone to form α-helical structure while interacting with other peptide chains in CFTR, and phosphorylation at S768 may destabilize the helix formation.

**Figure 4:**
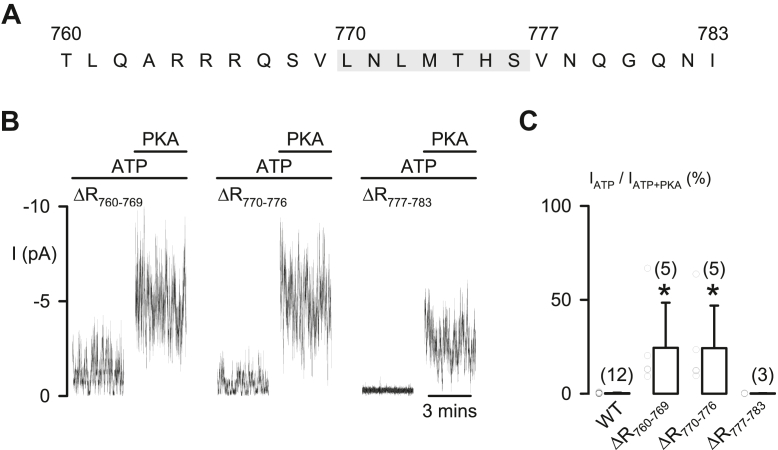
**Protein segments R**_**760–769**_**and R**_**770–776**_**contain inhibitory residues**. *A*, the diagram shows boundaries of the designated protein segments R_760–769_, R_770–776_, and R_777–783_ in R_760–783_. The *gray background* marks the sequence of R_770–776_. *B*, representative macroscopic CFTR Cl^−^ currents (I) of the indicated deletion mutants at 1 mM ATP before and after adding PKA (75 nM). *C*, fraction (%) of the CFTR Cl^−^ current in 1 mM ATP compared with that in ATP + PKA (=100%) for the tested deletion mutants. Data are means + SD with *circles* representing individual data points as independent biological replicates; numbers in *parentheses* indicate n; ∗*p* < 0.05 compared with the data of WT CFTR (obtained from Fig. 1*F*), one-way ANOVA with Dunn’s test. CFTR, cystic fibrosis transmembrane conductance regulator.

**Figure 5:**
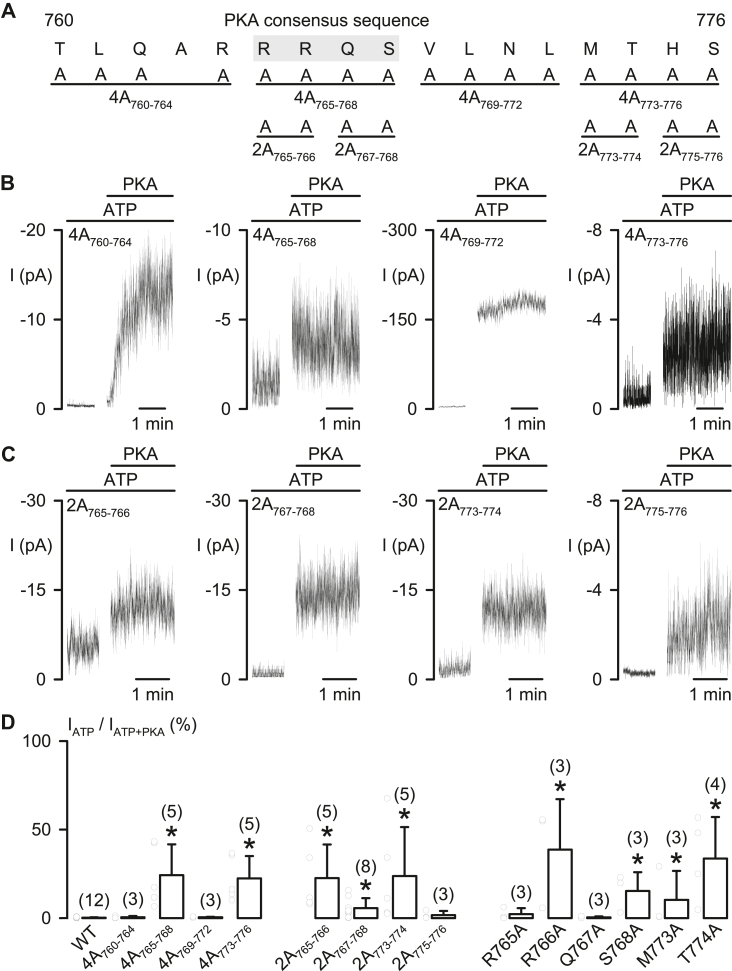
**Residues R^766^/S^768^ in the PKA consensus sequence at R**_**765–768**_**and M^773^/T^774^ block CFTR activation**. *A*, the diagram shows the positions of the PKA consensus sequence (R_765–768_; identified by *gray background*) and the amino acid residues that were replaced in mutant CFTRs by four alanine residues, named 4A mutations, including 4A_760–764_, 4A_765–768_, 4A_769–772,_ and 4A_773-776_, and those replaced with two alanine residues, termed 2A mutations, including 2A_765–766_, 2A_767–768_, 2A_773–774,_ and 2A_775–776_. *B* and *C*, representative macroscopic CFTR Cl^−^ currents (I) of the indicated 4A (*B*) and 2A (*C*) CFTR mutations in 1 mM ATP before and after adding 75 nM PKA. *D*, fraction (%) of the CFTR Cl^−^ current in 1 mM ATP compared with that in ATP + PKA (=100%) for the indicated alanine substitution mutants. Data are means + SD with *circles* representing individual data points as independent biological replicates; numbers in *parentheses* indicate n; ∗*p* < 0.05 compared with the data of WT CFTR (obtained from Fig. 1*F*), one-way ANOVA with Dunn’s test. CFTR, cystic fibrosis transmembrane conductance regulator.

Because R_763–777_ has high propensity to form the α-helical structure (19), the N771P mutation was used for breaking the formation of this long-length α-helix by the introduced proline residue (30). The data demonstrate that N771P failed to induce apparent constitutive current at 1 mM ATP (Fig. 3*I*), suggesting that the inhibition of R_760–783_ on CFTR activation may be originated by side-chain interactions of specific residues, rather than the contact of the whole α-helix (15). To explore the inhibitory sites, mutants with short-length peptide deletions (Fig. 4) or alanine replacement of one, two, or four residues within R_760–783_ (Fig. 5) were examined.

### Residues R^766^/S^768^ and M^773^/T^774^ in R_760–783_ prevent CFTR activation

By dividing R_760__–__783_ into three smaller protein segments (Fig. 4*A*), we found that deletion of segments R_760–769_ or R_770–776_, but not R_777–783_, generated constitutive CFTR Cl^−^ currents in the presence of 1 mM ATP (Fig. 4, *B* and *C*), similar to that of ΔR_760–783_-CFTR (Fig. 1, *D* and *F*). To avoid structural constraints created by deletion of a protein segment (10), we also simultaneously replaced four residues with alanine in R_760–776_ (4A mutations, Fig. 5, *A* and *B*) of wildtype CFTR to remove side-chain interactions of selected residues.

The mutations 4A_765–768_ and 4A_773–776_, but neither 4A_760–764_ nor 4A_769–772_, produced large PKA-independent constitutive CFTR Cl^−^ currents (Fig. 5, *B* and *D*). Using 2A mutations to screen residues in R_765–768_ and R_773–776_ (Fig. 5*A*), we identified the inhibitory residues 2A_765–766_, 2A_767–768_, and 2A_773–774_ that generated constitutive CFTR Cl^−^ currents (Fig. 5, *C* and *D*). Finally, single alanine substitutions demonstrated that four mutations, R766A, S768A, M773A, and T774A, elicited apparent constitutive CFTR Cl^−^ currents (Fig. 5*D*). This approach identified four residues, including R^766^/S^768^ in a PKA consensus sequence R^765^/R^766^/Q^767^/S^768^ (R_765–768_) (2) and M^773^/T^774^. The data strongly suggest that these four residues may form an inhibitory module that prevents CFTR activation by the side-chain interactions.

**Figure 6:**
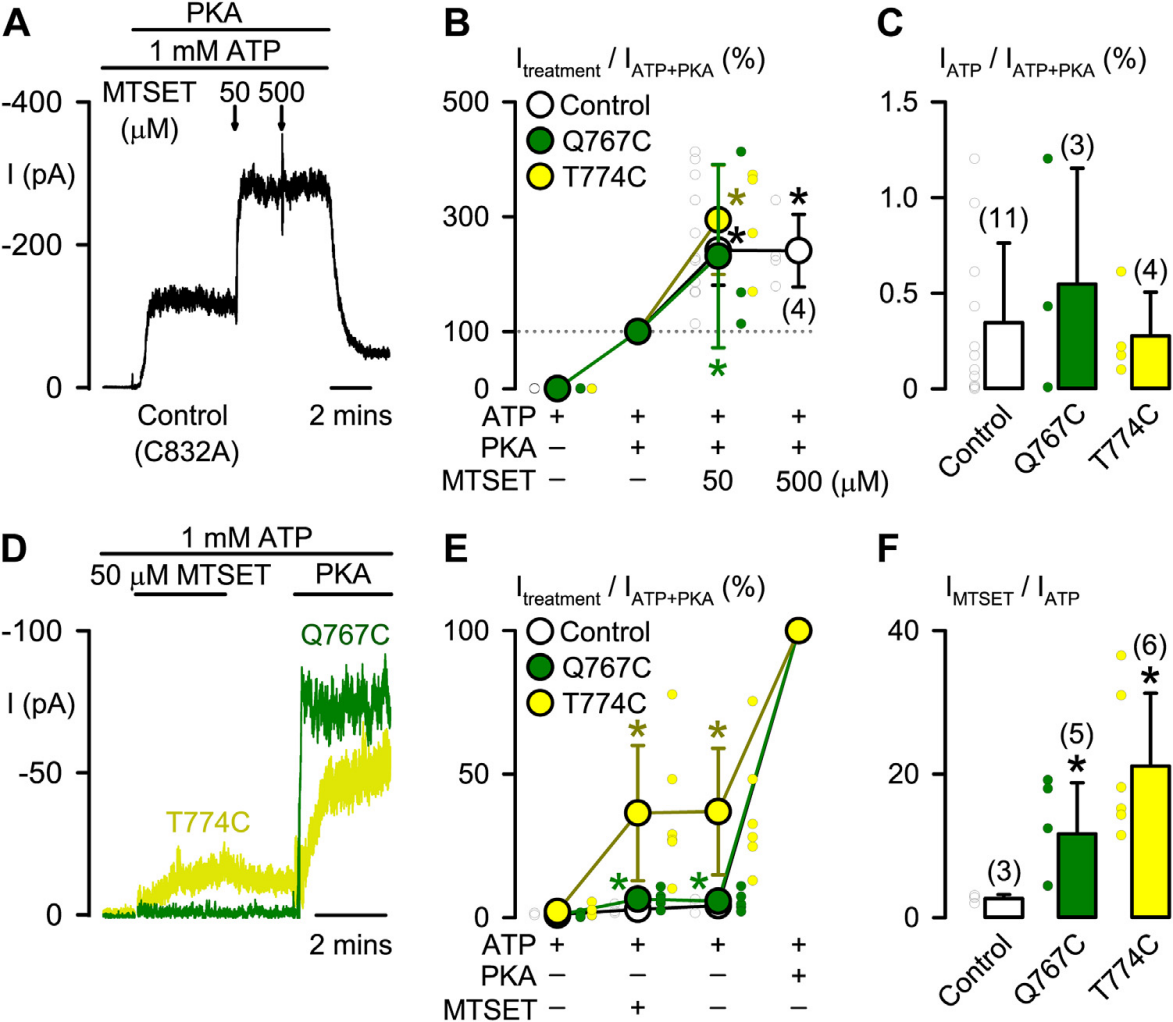
**MTSET modification of cysteine-substituted Q767C- and T774C-CFTR elicits PKA-independent constitutive channel activity**. *A* and *B*, effects of the cysteine modification reagent MTSET on the macroscopic CFTR Cl^−^ currents (I) of the control C832A-CFTR and two other mutants Q767C- and T774C-CFTR in the presence of ATP (1 mM) + PKA (75 nM). All data in this figure (*B* and *C*, *E* and *F*) are means ± SD with *circles* representing individual data points as independent biological replicates; SD bars are smaller than symbol size except where shown; the number in the parenthesis indicates n for the data at 500 μM MTSET; ∗*p* < 0.05 compared with the data of ATP + PKA (=100%) for each mutant CFTR, one-way ANOVA with Holm–Sidak test. *C*, fraction (%) of the CFTR Cl^−^ current in 1 mM ATP compared with that in ATP + PKA (=100%) for the tested CFTR mutants. Data are adopted from the condition of 1 mM ATP only of the three groups in *B*, numbers in *parentheses* indicate n. *D*–*F*, effects of MTSET on the macroscopic CFTR Cl^−^ currents of three mutant CFTRs in 1 mM ATP before 75 nM PKA was added. *D*, the time courses of Q767C- and T774C-CFTR Cl^−^ currents (I) with indicated treatments are shown. *E*, the percentage ratios (%) of mutant CFTR Cl^−^ currents with the indicated treatments (I_treatment_) compared with those in the presence of ATP + PKA (I_ATP__+__PKA_ = 100%) are shown. SD bars are smaller than symbol size except where shown. ∗*p* < 0.05 compared with the data of ATP + PKA (=100%) for each mutant CFTR, one-way ANOVA with Holm–Sidak test. *F*, the fold increase in mutant CFTR Cl^−^ current in the presence of ATP + MTSET (I_MTSET_) compared with that in 1 mM ATP (I_ATP_) is shown. Numbers in *parentheses* indicate n; ∗*p* < 0.05 compared with the control, one-way ANOVA with Dunn’s test. CTFR, cystic fibrosis transmembrane conductance regulator; MTSET, 2-(trimethylammonium)ethylmethanethiosulfonate.

To test this hypothesis, the mutations Q767C and T774C were engineered into the C832A-CFTR construct, which serves as a control (Fig. 6*A*). The C832A mutation may remove gating stimulation by cysteine modification at C^832^, such as that by *N*-ethylmaleimide attachment (31). Q767C is expected to have no impact on PKA phosphorylation at S^768^ (32) whereas T774C replaces threonine with the structurally similar residue cysteine. In the cysteine modification experiment, we assumed that the attached methanethiosulfonate compound at the mutated site Q767C may disturb side-chain interactions of R^766^/S^768^ and at T774C affecting that of M^773^ and engineered C^774^.

### Disruptions of side-chain interactions near or at the inhibitory residues partially activate the CFTR Cl^−^ channel

Positively charged 2-(trimethylammonium)ethylmethanethiosulfonate (MTSET) and negatively charged 2-(sulfonatoethyl)methanethiosulfonate (MTSES) are cysteine-modifying compounds (33), used to directly disrupt side-chain interactions around engineered cysteine residues. At 1 mM ATP, MTSES (50 μM) potently and irreversibly inhibited the channel activity of Q767C-C832A-, T774C-C832A-, and C832A-CFTR (N = 3 for each mutant, data not shown). MTSES was therefore abandoned as a probe of side-chain interactions, and subsequent experiments were performed using MTSET.

After adding PKA to activate the control construct C832A-CFTR (Fig. 6*A*), the CFTR Cl^–^ current was enhanced ∼2.4-fold by MTSET (50 μM), but no further enhancement of current was achieved by MTSET (500 μM) (Fig. 6, *A* and *B*). MTSET (50 μM) elicited similar stimulation of Q767C- and T774C-CFTR Cl^–^ currents (Fig. 6*B*). Moreover, these three CFTR constructs exhibited little CFTR Cl^–^ current before PKA addition (Fig. 6*C*). These data suggest that the Q767C and T774C mutations are without effect on MTSET-mediated stimulation of channel activity and do not prevent blockage of CFTR activation by the inhibitory module.

Adding MTSET prior to PKA increased the tiny current of C832A-CFTR ∼2.9-fold (Fig. 6, *E* and *F*), similar to its stimulatory effect on the fully activated channel (Fig. 6, *A* and *B*). The magnitude of CFTR Cl^–^ current was unaltered by later washout of MTSET but was markedly enhanced by PKA addition (Fig. 6*E*). These data suggest that MTSET is a strong potentiator of CFTR but itself is unable to activate C832A-CFTR.

By contrast, in the presence of 1 mM ATP, MTSET increased the magnitude of Q767C- and T774C-CFTR Cl^–^ current by ∼11.7- and ∼21.1-fold, respectively (Fig. 6, *D–F*), to achieve current levels noticeably larger than that of the control (Fig. 6*F*). The PKA-independent and MTSET-induced constitutive Cl^–^ current of Q767C- and T774C-CFTR was 6% and 36% of that after adding PKA (=100%, Fig. 6*E*), respectively. These data are comparable to that of 2A_767–768_- and S768A-CFTR (Fig. 5*D*) for Q767C and that of ΔR_760–783_-, 2A_773–774_-, and T774A-CFTR (Figs. 1*F* and 5*D*) for T774C. These data support the notion that the inhibitory module comprising R^766^/S^768^ and M^773^/T^774^ may block CFTR activation by side-chain interactions of the residues.

**Figure 7:**
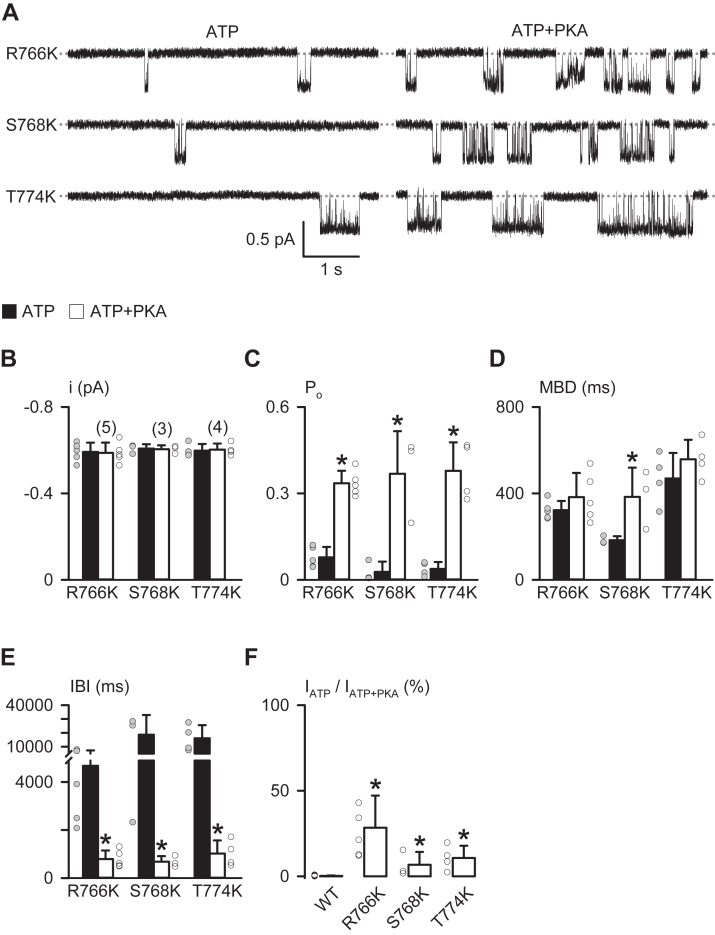
**R766K, S768K, andT774K mutations induce the constitutive single-channel activity of CFTR before PKA phosphorylation**. *A*, representative recordings show the single-channel activity of R766K-, S768K-, and T774K-CFTR in the presence of either ATP (1 mM) or ATP + PKA (75 nM). Each trace is 5 s long. The *dotted gray lines* indicate where channels are closed, and downward deflections represent channel openings. *B*–*E*, effects of PKA phosphorylation on the single-channel current amplitude (i), P_o_, MBD, and IBI of the indicated mutants. Columns are means + SD, and *circles* represent individual data points as independent biological replicates. Numbers in *parentheses* in *B* indicate n for *B*–*F*; ∗*p* < 0.05 compared with the data obtained at 1 mM ATP (*filled columns*) for each mutant, Student’s paired *t* test. *F*, fraction (%) of the CFTR Cl^−^ current (I) in 1 mM ATP compared with that in ATP + PKA (=100%) for the indicated mutants. Data of three mutants were derived by the equation I = N × i × P_o_, where N is the number of active channels, as described previously (50). Data are means + SD with *circles* representing individual data points as independent biological replicates. ∗*p* < 0.05 compared with the data of WT CFTR (obtained from Fig. 1*F*), one-way ANOVA with Dunn’s test. CTFR, cystic fibrosis transmembrane conductance regulator; IBI, interburst interval; MBD, mean burst duration.

To mimic the MTSET modification, S768K, R766K, and T774K mutations were individually constructed into wildtype CFTR (Fig. 7). Their single-channel activity was readily seen at 1 mM ATP in the absence of PKA (Fig. 7*A*). Similar to the result from the R domain deletion mutants (Fig. 2), adding PKA greatly increased the channel activity (Fig. 7*A*) and P_o_ (Fig. 7*C*) and largely decreased the IBI (Fig. 7*E*) of three CFTR mutants, but PKA was without effects on the single-channel current amplitude (i) (Fig. 7*B*) and MBD (Fig. 7*D*), except for an increase in the MBD of S768K-CFTR (Fig. 7*D*). Between three mutants, T774K-CFTR displayed MBD longer than that of other two mutants at 1 mM ATP and that of R766K-CFTR after adding PKA (*p* < 0.05, one-way ANOVA with Dunn’s test, Fig. 7*D*). Importantly, three mutants showed constitutive CFTR Cl^–^ current evidently before PKA was added (Fig. 7*F*), consistent with the result of MTSET modification experiments (Fig. 6*F*).

An alternative explanation is that aforementioned site-directed mutations (Fig. 7*F*) and MTSET attachment (Fig. 6*F*) might induce constitutive PKA-independent Cl^‒^ current by directly stimulating CFTR activity. On the contrary, the data of deletion mutations (Figs. 1 and 4) and alanine replacement (Fig. 5) suggest that the inhibitory interactions of R_760–783_ blocks CFTR activation. Recent studies also indicate that R_760–783_ has no stimulatory effect on channel gating after PKA phosphorylation (13) and the unphosphorylated R domain docks inside the intracellular vestibule between two NBDs for precluding channel opening (7). Thus, the best interpretation of our data in Figures 6 and 7 is that disrupting side-chain interactions of the inhibitory residues in R_760–783_ relieves the blockage of CFTR activation.

## Discussion

This study using deletion mutations revealed that R_760–783_ blocks CFTR activation (Figs. 1 and 2), as described previously (14). Since the constitutive Cl^–^ current of ΔR_760–783_-CFTR is not associated with intracellular phosphorylation by other kinases (14), here, we explored an autoinhibitory mechanism of R_760–783_ on channel activation. Studies of macroscopic and single-channel CFTR Cl^–^ currents (Figs. 1 and 2) demonstrated that R_760–783_ prevented channel activation, whereas R_708–759_ partially blocks the activation of ΔR_760–835_-CFTR. As R domain peptides (18, 19, 20) and the R_760–783_ segment (Fig. 3, *B* and *C*) are mostly unstructured, side-chain interactions of residues, rather than the interface contact of secondary structures, may mediate the blocking effects of R_760–783_ on CFTR activation.

S^768^, the only PKA phosphorylation site in R_760–783_, is the first site phosphorylated by PKA in purified R domain protein (17). Our work indicates that R^766^ and S^768^ within the PKA consensus sequence (R_765–768_) and M^773^/T^774^ together form an inhibitory module that prevents CFTR activation (Figure 4, Figure 5, Figure 6, Figure 7). However, the inhibitory interactions of these four residues seem weak but crucial for preventing channel activation, as residue deletion (Figs. 1, 2 and 4), replacement (Figs. 5 and 7), or modification (Fig. 6) were sufficient to induce partial channel activity before the addition of PKA. Because phosphorylated R_760–783_ has a minor role in the regulation of channel gating (Fig. 2) (13), the inhibitory module may prevent channel activation simply by avoiding conformational changes required for initiating channel gating.

The PKA dependence of ΔR_760–835_-CFTR (Figs. 1*F* and 2) revealed that R_708–759_ segment may block CFTR activation after deletion of R_760–783_ and R_784–835_. Moreover, our previous study indicates that phosphorylated R_708–759_ appears to inhibit IBI shortening (13), suggesting that the small PKA-induced decrease in the IBI of ΔR_760–835_-CFTR (Fig. 2*E*) may be caused by relieving the inhibition of unphosphorylated R_708–759_ on IBI shortening, rather than by the stimulatory effect of phosphorylated R_708–759_. Moreover, PKA phosphorylation at S^737^ in R_708–759_ is thought to be inhibitory to CFTR activation as the S737A mutation decreases the K_A_ of IBMX-dependent CFTR activation (25). It is also possible that unphosphorylated S^737^ directly inhibits channel activation. Further investigation is required for testing the effect of R_708–759_ on CFTR activation when R_760–783_ and R_784–835_ are present.

Conversely, the inhibitory effect of R_784–814_ on the frequency of channel opening by R_815–835_ (13) appeared to be strong, as the channel activity and IBI shortening of ΔR_760–783_- and ΔR_708–783_-CFTR were markedly PKA dependent (Fig. 2, *A*, *C* and *E*). Therefore, after removing the crucial blockage from the inhibitory residues in R_760–783_, large variations in the current ratio I_ATP_/I_ATP__+__PKA_ (Figs. 1*F* and 4*C*; 5*D*, 6*E* and 7*F*) may be due to the extent of the relief of primary inhibition on R_815–835_ (13) and then the relief of the inhibition by R_708–759_ (Fig. 2, *B* and *E*). We speculate that conformational fluctuations of the protein (34) might variously weaken the aforementioned inhibition by R domain segments.

**Figure 8:**
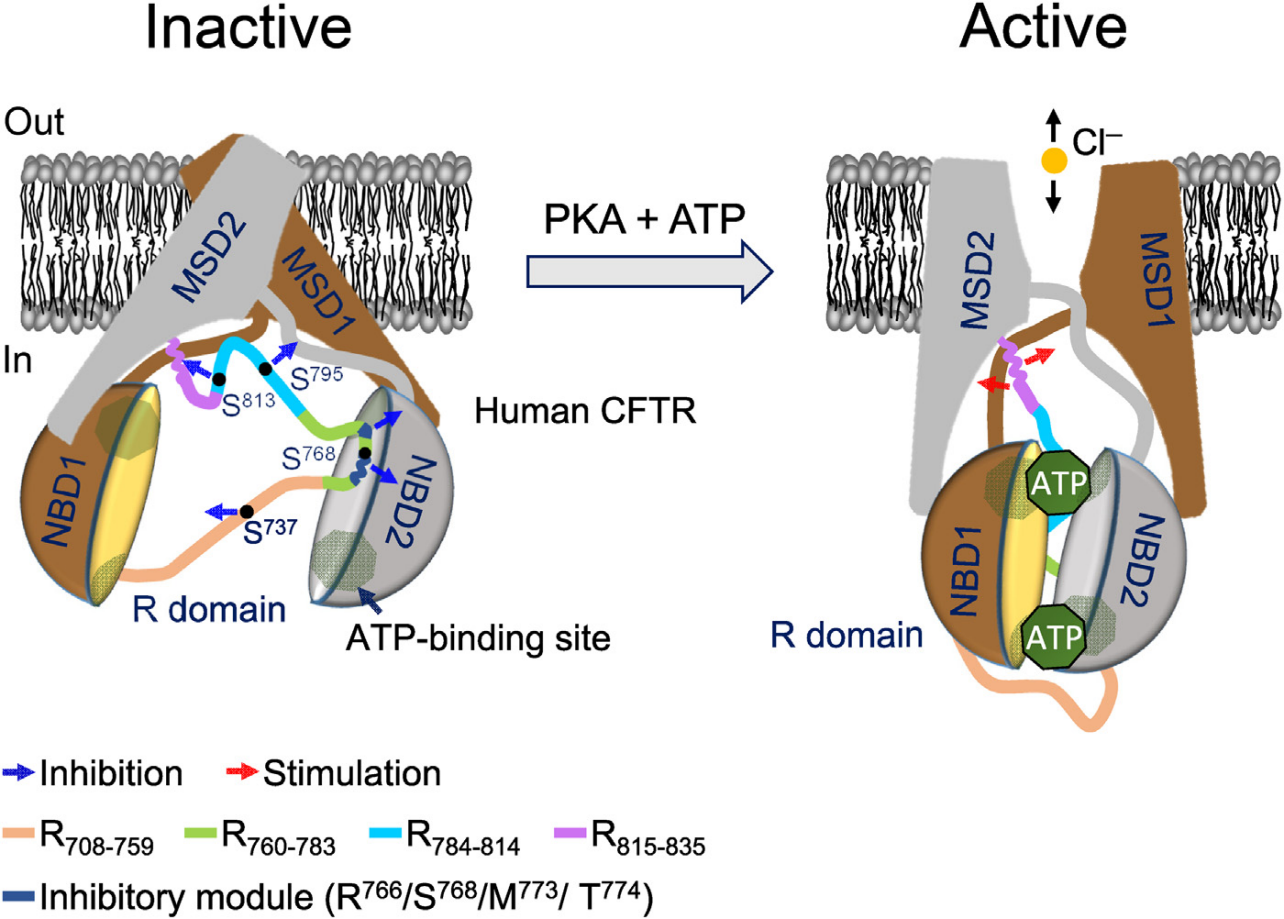
**The R domain controls phosphorylation-dependent CFTR activation**. The diagram shows proposed mechanism of CFTR inhibition and stimulation by the R domain before and after PKA phosphorylation. PKA consensus sequences that include S^737^, S^768^, S^795^, and S^813^ are presumed to inhibit CFTR activation. The interactions of the R domain segments with other domains of CFTR are assumed according to the recent cryo-EM structure (7). Inactive CFTR is blocked by three inhibitory segments (*blue arrows*), R_708–759_ including the phosphorylation site S^737^, R_760–783_ with an inhibitory module R^766^/S^768^/M^773^/T^774^ dominantly blocking channel activation as demonstrated in this study, and R_784–814_ with S^795^ and S^813^ inhibiting the stimulatory effect of R_815–835_ (13). Adding ATP and PKA phosphorylates CFTR and causes a large conformational change of the R domain (7), leading to CFTR activation with relieved R_815–835_ greatly stimulating channel gating (*red arrows*) (13). This two-step activation mechanism is an update from previous work (13). See text for details. CTFR, cystic fibrosis transmembrane conductance regulator.

Taken together, this study and our previous work (13) demonstrate a two-step activation mechanism of CFTR by PKA phosphorylation (Fig. 8). The first step (*orange and green segments*, Fig. 8) includes the removal of the dominant blocking effect by the inhibitory module in R_760–783_ (Figure 5, Figure 6, Figure 7) and the elimination of the additional restriction by R_708–759_ (Fig. 2, *B* and *E*). The second step (*blue and purple segments*, Fig. 8) involves relief of R_784–814_-mediated autoinhibition on R_815–835_, followed by strong stimulation of channel gating by R_815–835_, as described previously (13). It remains unclear whether the R domain interacts with only NBD1 or NBD2 or both.

Structurally disordered R_760–783_ (Fig. 3, *B* and *C*) may provide a large number of contacts and a long capture radius for the binding site per residue, as suggested by the fly-casting mechanism of unstructured protein molecules (35), for facilitating fast PKA-dependent on–off inhibitory interactions, which modulate and match CFTR activation with intracellular cAMP signaling (6, 36).

The proposed PKA-dependent CFTR activation mechanism (Fig. 8) is consistent with previous findings that CFTR activation follows sequential PKA phosphorylation at the R domain (17, 37, 38) possibly starting at S^768^ (17). The first and second activation steps in the mechanism may represent the inhibitory (11, 37) and stimulatory regulation (12, 13) of CFTR activity by the R domain, respectively.

### R_708–759_ and R_760–783_ protein segments block CFTR channel opening

The similar gating kinetics of ΔR_760–783_- and ΔR_708–783_-CFTR before PKA addition (Fig. 2, *C*–*E*) indicates that R_708–759-_ and R_760–783_-mediated restriction of channel activity is not a graded inhibition of CFTR activity but an on–off regulation of N, the number of active channels. One caveat of these studies is whether N would be underestimated because of low P_o_ (Fig. 2, *B* and *C*). We therefore calculated the recording time required to observe two simultaneous channel openings in a membrane patch using the equation: (3‧τ_o_/N)/(P_o_)^N^ (39), where the open time τ_o_ can be estimated from MBD values. For ΔR_760–835_-CFTR with P_o_ = 0.06 and MBD = 258 ms before PKA addition (Fig. 2, *C* and *D*), 108 s is required to observe two active channels. Therefore, our recording time of 3 min at 1 mM ATP would be sufficient to distinguish membrane patches that contain one active channel from those with two or more active channels.

Due to low P_o_ values before PKA addition (Fig. 2*C*), it is possible that we might have underestimated the N of ΔR_760–835_-CFTR (Fig. 2*B*) in few experiments. In these instances, the blocking action of R_708–759_ would likely be less effective, but its regulatory effect on the IBI shortening would be stronger than that suggested by our data (Fig. 2*E*).

Studies on ΔR_708–835_/S660A-CFTR (12, 13, 37, 40) and split CFTRs (10) indicate that with the most R domain deleted, channel gating can be functionally operated in the presence of ATP without PKA. Interestingly, R_815–835_ that contains no PKA phosphorylation sites plays an important role in stimulating channel opening (13) and the rest of the R domain R_708–759_ and R_760–783_ after PKA phosphorylation show minor effects on channel gating and R_784–814_ has moderate effects on MBD (13). Moreover, phosphorylated peptides R_709–738_, R_733–754,_ and R_760–784_ show no stimulation on the channel activity of ΔR_708–835_/S660A-CFTR (13). These data suggest that R_708–759_ and R_760–783_ prevent CFTR activation by blocking channel activity, rather than generating the gating stimulation after PKA phosphorylation. Consistently, the R domain in the cryo-EM structure moves away from the interface of two NBDs after PKA phosphorylation (7).

Of note, the binding of ATP or other nucleotides to the NBDs seems independent of PKA phosphorylation (41, 42). Therefore, ATP-induced NBD dimerization (8) would be the key step for R_708–759_ and R_760–783_ to prevent channel opening. This idea is supported by evidence that PKA phosphorylation is required for NBD dimerization (43) and the ATPase activity of the NBDs (44). Thus, the PKA-dependent elongation on the MBD of S768K-CFTR (Fig. 7*D*) may suggest that S768K only partially relieved the blockage of CFTR activation by the inhibitory module before PKA phosphorylation. Moreover, in the cryo-EM structure of CFTR (7), R_825–843_ engages with intracellularly located residues of transmembrane segments 9, 10, and 12 of MSD2. It is speculated that R_760–783_ and R_708–759_ located more than 40 amino acid residues from R_825–843_ may be capable of interacting with the two NBDs to prevent their dimerization (7, 22).

### Residues of the inhibitory module preclude CFTR activation

The sequence alignment of R_765–774_ (Table 1) shows that this protein segment is highly conserved among different CFTR orthologs (45), especially for the four residues, R^766^, S^768^, M^773^, and T^774^, in the inhibitory module. The NMR study indicates that the short segment R_763–777_ contains the most stabilized α-helical structure in the R domain (19). Our data suggest that when induced by TFE, the R_760–783_ peptides may form α-helical structure with a plateau of helicity at the concentration around 30 to 40% (Fig. 3*H*), as seen in other peptides with amino acid sequences predicted to form helical structures (28). At or below these concentrations, TFE facilitates α-helix formation primarily by promoting TFE–H_2_O exchange on the peptide (28, 29), suggesting that phosphorylation at S^768^ might promote water retention on the surface of the peptide to destabilize its helical structure.

**Table 1:** Sequence alignment of CFTR amino acid residues 765–774 from various species

Species	765	R domain residues								774	Sequence ID
Human	R	R	Q	S	V	L	N	L	M	T	gb|AAA35680.1
Chimpanzee	R	R	Q	S	V	L	N	L	M	T	gb|AAM08348.1|AF490140_1
Lemur	R	R	Q	S	V	L	N	L	M	T	sp|Q2QL83.1|CFTR_MICMU
Monkey	R	R	Q	S	V	L	**M**	L	M	T	sp|Q09YK5.1|CFTR_ATEGE
Naked mole-rat	R	R	Q	S	V	L	N	L	M	T	gb|EHB03919.1
Bat	R	R	Q	S	V	L	N	L	M	T	gb|ABC87480.1
Duck	R	R	Q	S	V	L	N	L	M	T	gb|EOA97656.1
Frog	R	R	Q	S	V	L	N	L	M	T	gb|AAC60023.1
Zebrafish	R	R	Q	S	V	L	**A**	**F**	M	T	NP_001038348.1
Pufferfish	R	R	Q	S	V	L	**A**	**F**	**I**	T	NP_001041505.1
Sea lamprey	R	R	**K**	S	V	L	**H**	L	M	**L**	MN216027.1

The residues with *underlines* indicate the PKA consensus sequence RRQS and two residues, M^773^ and T^774,^ in human CFTR and those labeled in *bold* identify distinct amino acids found at the same positions in different CFTR orthologs. Sequence IDs are adapted from the database of the National Center for Biotechnology Information Reference Sequence.

Moreover, interactions of bioactive peptides with their receptors may create a hydrophobic environment, mimicking TFE-induced effects (28), leading to a structural alteration from random coil to α-helix (46). We speculated that the short-length helical structure may align R^766^/S^768^ with M^773^/T^774^ (α-helix, 3.6 residues/turn) to face a similar direction for blocking CFTR activation together, so that either the deletion or residue replacement of R^766^ or T^774^ displayed similar levels of constitutive CFTR Cl^−^ current (Figs. 1, 4, 5 and 7).

### R^766^ and S^768^ in the PKA consensus sequence R_765–768_ inhibit CFTR activation

Early studies suggest that phosphorylation at S^768^ is inhibitory to PKA-dependent CFTR activation and channel gating (17, 25, 26) because the S768A mutation reduces the half-maximal activation concentration of 3-isobutyl-1-methylxanthine (25) and decreases the IBI, the closed time between channel openings (17, 26). However, ΔR_760–783_ shows little effect on CFTR channel gating (Fig. 2, *C*–*E*) (13), suggesting that S768A-induced stimulation of CFTR activity may be derived from new interactions of the unphosphorylated peptide.

Evidence shows that interactions between S^768^ and H^950^ may play a role in precluding CFTR activation (16), whereas the mutations S768D (16), S768A (Fig. 5*D*) (16) and S768K (Fig. 7*F*) only induce modest PKA-independent CFTR Cl^−^ currents. By contrast, this study revealed that R^766^, like T^774^, serves as an important inhibitory residue (Figs. 5*D* and 7*F*). Due to the large level of constitutive current induced by R766K (Fig. 7*F*) and the high structure similarity between two residues arginine and lysine except for the shorter side chain of lysine, the data suggest that precise side-chain interactions, rather than surface positive charge at R^766^, are crucial for blocking CFTR activation. Therefore, we speculate that the process of PKA binding to the consensus sequence (47) at R_765–768_ in the R domain may be sufficient to remove the restriction of channel activation by the inhibitory module, consistent with a recent study (9) and phosphorylation at S^768^ may stabilize the unbound conformation of the peptide.

The complex effects of R766K, S768K, and T774K on CFTR gating kinetics (Fig. 7, *C*–*E*) reveal that the replacement of these inhibitory residues may disturb conformational changes or peptide–peptide interactions associated with ATP-dependent channel gating. The underlying mechanism requires further explorations as the residue substitution may mimic or disrupt the side-chain interactions of original residues but may possibly also create new interactions affecting channel gating.

It is of note that several limitations in this study may weaken our interpretations to the results. First, the effects of R_708–759_ on CFTR activation and gating regulation were only observed in ΔR_760–835_-CFTR that deletes protein segments R_760–783_ and R_784–835_. In addition to the dominant inhibitory effect of R_760–783_ as shown in this study, R_784–835_ also weakly inhibits CFTR activation (*e*.*g*., ΔR_708–783_-CFTR, Figs. 1*F* and 2*B*), partly because of R_784–814_ precluding the gating stimulation by R_815–835_ (13). Therefore, the inhibitory effect of R_708–759_ in other deletion mutants in Figures 1 and 2 may be overshadowed by the action of the remaining R_760–783_ and R_784–835_ segments. Conversely, R_708–759_ in ΔR_760–835_-CFTR could interact with a region different from the original position in wildtype CFTR but generate interdomain interactions that functionally mimic the effects of R_760–783_ or R_784–835_. The deletion mutations may cause other confounding factors, such as constrained peptide (10).

Second, this study assessed the inhibition of R_708–759_ by measuring changes in the number of active channels before and after adding PKA, whereas because of technical limitations, short recording time, small numbers of channels per membrane patch, and few experiments may reduce data quality. Third, the correlation between TFE-induced helical structure (Fig. 3*H*) and interdomain interactions of the R domain segments has not been further explored in this study. Future investigations are required to elucidate how side-chain interactions of the inhibitory module can prevent CFTR activation and how this inhibition is relieved during the process of PKA phosphorylation, such as PKA binding (9) and the attachment of the phosphate group (37).

### Closing remarks

This study and our previous work (13) shed a light on a long-standing puzzle why the R domain can generate both stimulatory and inhibitory effects on CFTR activity (3) (Fig. 8). This study also identified a cluster of four residues, R^766^/S^768^ and nearby M^773^/T^774^, forming an inhibitory module that precludes CFTR activation. Such an inhibitory mechanism combining residues in and around a PKA consensus sequence might be emulated by other PKA-dependent proteins.

## Experimental procedures

### Mutagenesis and CFTR expression

Human CFTR mutants were either constructed and expressed using the pTM1-CFTR4/S660A plasmid and the vaccinia virus/bacteriophage T7 hybrid expression system as described previously (13) or the pcDNA3.1 expression vector. CFTR mutants were made using the QuikChange II XL Site-Directed Mutagenesis Kit (Agilent Technologies) and verified by DNA sequencing. HeLa cells were cultured with Dulbecco's modified Eagle's medium (Gibco, Thermo Fisher Scientific) with 10% fetal bovine serum at 37 °C and 5% CO_2_. After 1 h infection with the vaccinia virus only for pTM1-CFTR plasmids, HeLa cells were transfected with wildtype or mutant CFTR plasmids using Lipofectamine 2000 (Invitrogen). After 12 to 48 h, transfected cells were used for patch-clamp experiments.

### Electrophysiology

The patch-clamp method was used as described previously (13, 40). In excised inside–out membrane patches, the channel activity of wildtype or mutant CFTRs was initially recorded in the presence of 1 mM ATP in the intracellular solution for >3 min and then activated by adding 75 nM PKA catalytic subunit, purified from bovine heart (Calbiochem), into the bath (intracellular) solution at room temperature. To amplify the magnitude of CFTR current, membrane voltage was clamped at −50 mV and a large Cl^−^ concentration gradient applied across the membrane patch. The pipette (extracellular) solution contained (millimolar): 140 *N*-methyl-d-glucamine, 140 aspartic acid, 5 CaCl_2_, 2 MgSO_4,_ and 10 tricine, pH 7.4 with Tris ([Cl^–^], 10 mM). The bath solution contained (millimolar): 140 *N*-methyl-d-glucamine, 3 MgCl_2_, 1 CsEGTA, 10 tricine, pH 7.4 with HCl.

Stock solutions of ATP were prepared fresh before each experiment. After ATP was dissolved in the intracellular solution, the pH of the intracellular solution was readjusted with NaOH (40). Stock solutions of 250 mM MTSET-Cl or Na-MTSES (Toronto Research Chemicals) were dissolved in water and stored at −80 °C.

CFTR Cl^−^ currents were recorded using an Axopatch 200B patch-clamp amplifier (Molecular Devices) and filtered with an 8-pole Bessel filter (Frequency Devices, Inc) at a corner frequency of 500 Hz and digitized with a Digidata 1322 interface (Molecular Devices) at 10 kHz. The software package pCLAMP 10 (Molecular Devices) was used for data acquisition and analysis. For the purpose of illustration, recordings were digitally filtered at 1 kHz for single-channel recordings and 50 Hz for macroscopic currents.

For membrane patches that contained many channels, macroscopic CFTR Cl^−^ currents were averaged for data analysis. For membrane patches that contained five or less channels, single-channel analysis was performed. The number of active channels (N) in a membrane patch was determined by the maximum number of channels that opened simultaneously at one time during the intervention (48). To measure the P_o_, event lists of open and closed times were created using a half-amplitude crossing criterion, whereas transitions with durations less than 1 ms were excluded.

To measure MBD, the cutoff time of 20 ms was used to separate the short closures within bursts of channel openings from the long closures separating bursts of channel openings (13). The IBI was calculated from P_o_, MBD, and P_o_-in-burst (P_o-burst_) using the equation below:

$$P_{o}=\frac{\text{MBD}\times P_{o-\text{burst}}}{\text{MBD}+\text{IBI}}$$

Because N might possibly be underestimated for mutant CFTRs with large gating anomalies, the reported P_o_ values of these CFTR mutants should be considered as maximal P_o_ values and their IBI values as minimal IBI values.

### CD measurement

Unphosphorylated R_760–783_ peptides and R_760–783_ peptides labeled by phosphorylation at S^768^ with a purity of >98.5% were synthesized by the Chinese Peptide Company. Synthesized peptides were dissolved in dilute HCl and dialyzed to remove the trifluoroacetate ions left from HPLC, forming the hydrochloride salt. The samples were lyophilized after dialysis and redissolved in nanopure water, prior to determining their concentration by amino acid analysis (AAA Service Lab). A JASCO 810 instrument was used for CD measurements with the following conditions: three scans were recorded and averaged from 190 to 260 nm; scan speed, 100 nm/min; bandwidth, 1.00 nm; and temperature 20 °C.

The measurements were made in a 1 mm quartz cuvette at a fixed peptide concentration of 20 μM. The buffer solution contained 20 mM Tris (pH 7.01) or 20 mM Tris–NaCl (30 mM NaCl, pH 7.01). The percentage of TFE corresponds to a volumetric amount to the Tris-buffered solution, as the blank. For data processing, a blank signal containing only solvent (*e*.*g*., buffer or buffer/TFE) was subtracted from signals obtained with peptide-containing samples. The helical content % was calculated using the molar ellipticity ([θ], expressed in deg⸳cm^2^/dmol) of −3000 for random coil and −39,500 for 100% helical, as previously described (49). TFE, Tris buffer, and NaCl were obtained from Fisher Scientific.

### Reagents and chemicals

Chemicals were purchased from the Sigma–Aldrich Corp and were of reagent grade.

### Statistics

Data are presented as means ± SD of n observations. By using the software SigmaPlot, statistical differences between sets of data were analyzed by Student’s paired *t* test or one-way ANOVA with Shapiro–Wilk test for normality and with either *post hoc* Holm–Sidak or Dunn’s test. Differences were considered statistically significant when *p* < 0.05.

## Data availability

All data are contained within the article.

## Conflict of interest

The authors declare that they have no conflicts of interest with the contents of this article.
